# A possible approach for gel-based proteomic studies in recalcitrant woody plants

**DOI:** 10.1186/2193-1801-2-210

**Published:** 2013-05-08

**Authors:** Mónica Sebastiana, Andreia Figueiredo, Filipa Monteiro, Joana Martins, Catarina Franco, Ana Varela Coelho, Fátima Vaz, Tânia Simões, Deborah Penque, Maria Salomé Pais, Sílvia Ferreira

**Affiliations:** Plant Systems Biology Lab, Center of Biodiversity, Functional & Integrative Genomics (BioFIG), Science Faculty of Lisbon University, Lisbon, 1749-016 Portugal; Instituto de Tecnologia Química e Biológica, Universidade Nova de Lisboa, Av. da Republica, Oeiras, 2780-157 Portugal; Laboratório de Proteómica, Departamento de Genética, Instituto Nacional de Saúde Dr. Ricardo Jorge INSA I.P, Lisbon, Portugal

**Keywords:** Grapevine, Pine, Oak, Ectomycorrhizal roots, Protein extraction, 2-DE, Mass spectrometry

## Abstract

Woody plants are particularly difficult to investigate due to high phenolic, resin, and tannin contents and laborious sample preparation. In particular, protein isolation from woody plants for two-dimensional gel electrophoresis (2-DE) is challenging as secondary metabolites negatively interfere with protein extraction and separation. In this study, three protein extraction protocols, using TCA, phenol and ethanol as precipitation or extraction agents, were tested in order to select the more efficient for woody recalcitrant plant gel-based proteomics. Grapevine leaves, pine needles and cork oak ectomycorrhizal roots were used to represent woody plant species and tissues. The phenol protocol produced higher quality 2-DE gels, with increased number of resolved spots, better spot focusing and representation of all molecular mass and isoelectric point ranges tested. In order to test the compatibility of the phenol extracted proteomes with protein identification several spots were excised from the phenol gels and analyzed by mass spectrometry (MALDI-TOF/TOF). Regardless the incomplete genome/protein databases for the plant species under analysis, 49 proteins were identified by Peptide Mass Fingerprint (PMF). Proteomic data have been deposited to the ProteomeXchange with identifier PXD000224. Our results demonstrate the complexity of protein extraction from woody plant tissues and the suitability of the phenol protocol for obtaining high quality protein extracts for efficient 2-DE separation and downstream applications such as protein identification by mass spectrometry.

**Electronic supplementary material:**

The online version of this article (doi:10.1186/2193-1801-2-210) contains supplementary material, which is available to authorized users.

## Background

Nowadays, proteomics constitutes one of the priority research areas in biological sciences. Knowledge generated in the past years has shown that dynamism, variability and behaviour of proteins are more complex than what was thought (Abril et al. 2011). Unlike model biological systems, the full potential of proteomics is far from being completely exploited in plant biology research. Thus, only a low number of plant species have been investigated at the proteomics level and, mainly, by using strategies based on 2-DE coupled to MS, resulting in low proteome coverage (Carpentier et al. 2008). On proteomics, most of the biological research has been carried on model plants such as *Arabidopsis thaliana*, *Solanum tuberosum* or *Medicago truncatula*. Yet, knowledge generated from these and other model plants need to be applied to other plant species. Within the plant group, woody species are the most difficult to investigate due to high phenolic, resin, and tannin contents, as well as, very often, an incompletely sequenced genomes. In the plant kingdom, woody species are found within both Angiosperms and Gymnosperms. On the Gymnosperm group, much research has been conducted on the genus *Pinus* (Wu et al. 2008;Valledor et al. 2008,2010;Wang et al. 2013), with Maritime pine (*Pinus pinaster* Ait.) being one of the most representative species used for reforestation in South-western Europe. Angiosperm considers a large variety of broad-leaved trees and shrubs including oak and grapevine. Grapevine (*Vitis vinifera*) is considered the most important fruit plant throughout the world, thus much proteomic research has been conducted in the last decade on this species (reviewed in Giribaldi and Giuffrida 2010). Cork Oak (*Quercus suber* L.) is a Mediterranean forest species with a remarkable ecological, social and economic value. Cork production from cork-oak supports an industry of economic and social relevance in Mediterranean countries, but few proteomic studies have been conducted (Gómez et al. 2009;Ricardo et al. 2011).

For proteomic studies, particularly in woody species, sample preparation and protein separation are of extreme importance for optimal results as most problems associated with 2-DE can be traced down to the co-extraction of non protein cellular components that affect protein gel migration. Plant tissues are very rich in proteases and interfering compounds such as secondary metabolites (Wang et al. 2008), thus comparatively to other organisms, extraction of proteins is of great challenge (Görg et al. 2004;Isaacson et al. 2006). Two protocols, TCA-acetone and phenol, are generally used with some optimization related to the specific tissue, in function of the amounts of indigenous contaminants (organic acids, lipids, polyphenols, pigments or terpenes among others). The TCA-acetone protocol was initially developed by Damerval et al. (1986) and is based on protein denaturation and precipitation under acidic/hydrophobic conditions, which help to concentrate proteins and remove contaminants (Wang et al. 2008). Up to date, this is the most used protocol for protein extraction from plant tissues for proteomic analysis (Jorrín et al. 2007;Jorrín-Novo et al. 2009). For recalcitrant tissues, the phenol-based method has the potential to generate samples of higher purity than TCA-acetone, as compounds such as polysaccharides and other water-soluble contaminants are separated from the proteins that are solubilized in the phenolic layer (Hurkman and Tanaka 1986).

Until now studies comparing protein extraction protocols for plant proteomics have been focused on herbaceous plants, mainly on fruit tissues (Saravanan and Rose 2004;Carpentier et al. 2005;Song et al. 2006;Zheng et al. 2007), with few being conducted on woody plant tissues (Jellouli et al. 2010;Dziedzic and McDonald 2012). With this study we aimed to compare three previously published protein extraction protocols and to evaluate their performance for the extraction of high-quality protein extracts suitable for 2-DE and MS analysis using woody recalcitrant plant tissues (leaves and roots). We have used pine needles representing a tissue that is highly rich in terpene metabolites (Wang et al. 2008); grapevine mature leaves, typically more problematic during 2-DE analysis than young leaves due to high levels of polyphenols and organic acids (Wang et al. 2008), and cork oak roots, a highly vacuolated with low protein content and high level of secondary metabolites such as lignin (Chatterjee et al. 2012). Moreover, cork oak roots typically establishes ectomycorrhizal (ECM) symbiosis and the symbiotic fungus may present triterpenoids and pigments (Baumert et al. 1997) that can also interfere with 2-DE. We have tested the two most commonly used protein extraction methods in plants, TCA-acetone (Damerval et al. 1986) and phenol (Hurkman and Tanaka 1986), as well as a single-step ethanol precipitation-based protocol that was successfully applied to poplar proteome isolation (Ferreira et al. 2006), in order to select the best extraction method for woody recalcitrant plant species/tissues. As mass spectrometry is one of the most used techniques for protein identification, compatibility of the best protein extraction method with mass spectrometry was tested.

## Results

**Figure 1:**
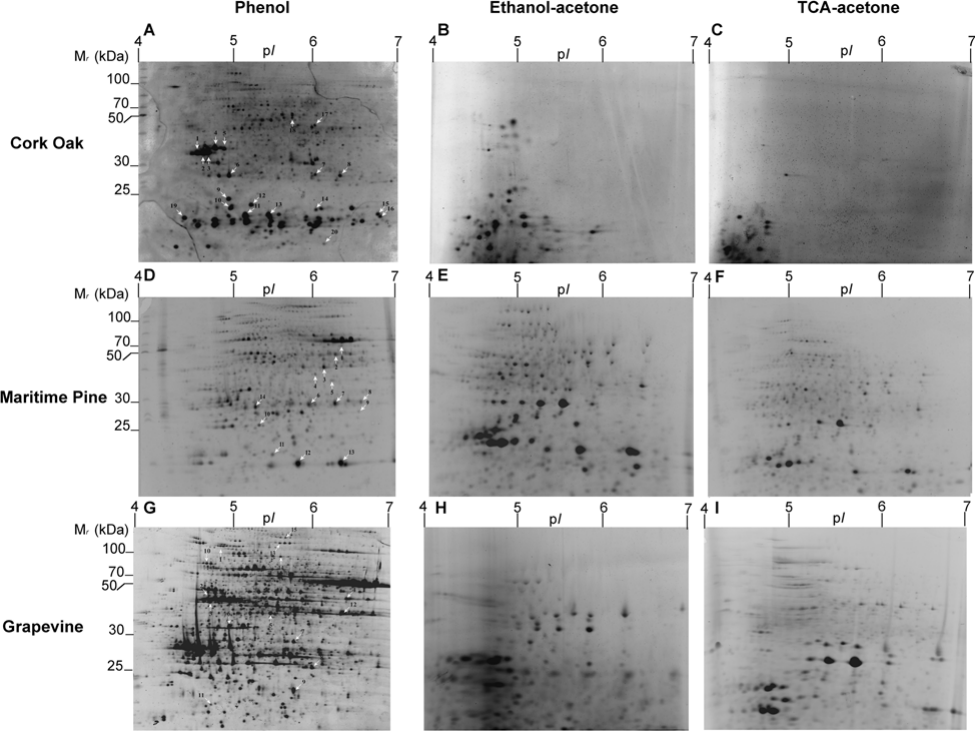
**Maritime pine (*Pinus pinaster*) needles and grapevine (A, D, G), ethanol-acetone (B, E, H) and TCA-acetone (C, F, I) extraction methods from cork oak ECM roots, Martime Pine (*Pinus pinaster*) needles and Grapevine (*Vitis vinifera*cv Regent) leaves.** Proteins were separated on a 4–7 linear pH gradient in the first dimension (IEF) and 15% polyacrylamide gels in the second dimension.

**Figure 2:**
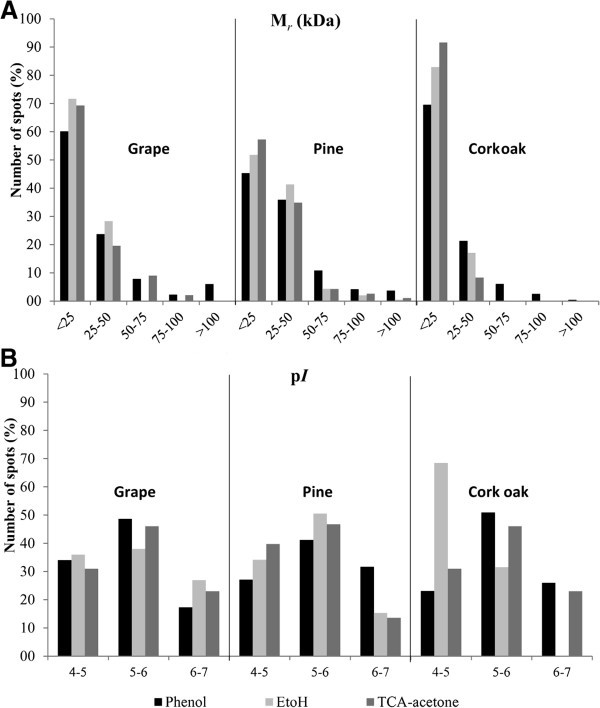
**2-DE distribution of protein spots from grapevine fully developed leaves, pine needles and cork oak ectomycorrhizal roots proteomes extracted in the three protocols tested, according to their M**_***r***_**(A) and p*I*(B).**

Considering the protein yield obtained with the different protocols, a similar trend was observed in the different species/tissues analysed: ethanol-acetone precipitation allowed obtaining higher amounts of protein (3.6 – 21.9 mg/g FW) than TCA-acetone precipitation (2.8 – 16.6 mg/g FW) and phenol-based extraction protocol (0.6 – 5.8 mg/g FW) (Table 1). Considering the amount of protein extracted from each plant material with the different extraction protocols, ECM oak roots produced the lowest protein yields (Table 1) with all the extraction protocols. For pine needles and grapevine leaves, the three protein isolation methods produced equivalent amounts of total protein. Representative 2-DE gels for each species/method are shown in Figure 1. Both qualitative and quantitative differences were found among 2-DE patterns for the three protein extraction protocols. For pine needles, all three extraction protocols resulted in good quality well-resolved gels (Figure 1D,E,F). However, when compared with the phenol protocol, TCA-acetone and ethanol-acetone have resulted in lower number of spots as well as reduced in several areas of the gels especially at the high molecular weight region particularly for the highest p*I* range. For grapevine leaves, the phenol protocol resulted in good quality gels with efficient protein separation and good spot focusing (Figure 1G). TCA and ethanol produced inferior quality gels, when compared to phenol, with decreased spot focusing and under representation of proteins in the high molecular mass area of the gels (Figure 1H,I). For ECM oak roots, the phenol protocol was the only producing high quality gels (Figure 1A), with TCA-acetone and ethanol extraction methods producing atypical gels with deficient protein separation, low number of protein spots and bad spot focusing (Figure 1B,C). The highest number of protein spots observed in gels was using the phenol extraction for all the three species/tissues analysed (532 – 904 spots) (Table 1). For grapevine leaves and pine needles, TCA-acetone resulted in an intermediate number of spots (657 and 362, respectively) and ethanol precipitation produced the lowest number of spots (166 and 392, respectively). In ECM oak roots, both TCA-acetone and ethanol produced a significantly lower amount of spots when compared with the phenol protocol (904), with ethanol producing 111 spots and TCA-acetone only 36 spots. To characterize quantitative differences between the protocols assayed, spot distribution by molecular mass and p*I* were compared for the three extraction methods (Figure 2). For all the plant tissues/species analysed the phenol protocol permitted to obtain a more evenly spot distribution across all M_*r*_ and p*I* regions. On the contrary, with the TCA-acetone and ethanol extraction protocols spots were located preferentially at the lower M_*r*_ and acidic p*I* regions of the gels, especially in ECM oak roots. The phenol extraction protocol permitted to obtain more spots within the high molecular mass range when compared with the other two precipitation methods.

**Table 1:** **Protein yields and total number of 2-DE protein spots, from grape leaves, pine needles, and cork oak ectomycorrhizal ECM roots after phenol, ethanol and TCA-acetone extraction protocols**

Plant species	Protocol	Protein yield (mg/g FW)^a^	Total number of spots
Pine	Phenol	5.81 ± 0.46	805
	Ethanol	21.88 ± 4.00	392
	TCA-acetone	13.86 ± 1.14	657
Grapevine	Phenol	3.78 ± 0.61	532
	Ethanol	20.55 ± 1.79	166
	TCA-acetone	16.57 ± 1.31	362
Oak	Phenol	0.61 ± 0.14	904
	Ethanol	3.57 ± 0.20	111
	TCA-acetone	2.77 ± 0.14	36

^a^ Mean and standard deviation from three technical replicates.

FW, fresh weight.

**Figure 3:**
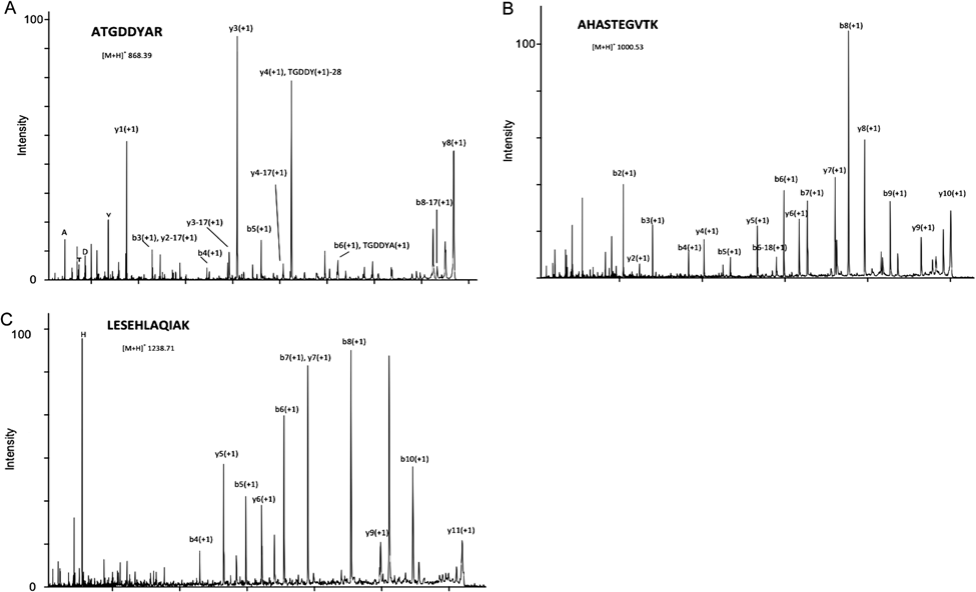
**Examples of tandem MS spectra of protein spots excised from a 2-DE gel, trypsin-digested and analyzed by MALDI-TOF/TOF.** (**A**) Spot S6 MS/MS spectrum of the parent ion [MH] + 1 868.39 identified as ATGDDYAR; (**B**) Spot P1 MS/MS spectrum of the parent ion [MH] + 1 1000.53 identified as AHASTEGVTK; (**C**) Spot V6 MS/MS spectrum of the parent ion [MH] + 1 1069.57 identified as LESEHLAQIAK.

As the phenol protocol was found to be the most adequate to extract proteins from the three species/tissues analysed, its compatibility with MS for protein identification was investigated. Several protein spots from the phenol 2-DE gels from each species/tissue were excised and identified by MS. Protein spots were chosen from different gels regions in order to include acidic, basic, high and low molecular mass proteins and also different spot intensities. MALDI-TOF/TOF analysis showed that excised protein spots lead to good quality spectra (Figure 3A,B,C). Results of protein identification by MALDI-TOF/TOF are presented in Table 2 and Additional file 1: Table S1, Additional file 2: Table S2, Additional file 3: Table S3. Of the 52 total spots analysed in the three species, all were identified with significant MOWSE/ProteinPilot scores (i.e., a score greater than 50/2, respectively, at p < 0.05) confirming the compatibility of the phenol extraction method with MS analysis.

**Table 2:** **Protein annotation in the grapevine fully developed leaves (V1-V15), cork oak ectomycorrhizal roots (S1-S20) and pine needles (P1-P14) spots excised from 2-DE gels and trypsin-digested**

Spot	Protein ID	Annotation	Score	Search engine	Protein score	Sequence of the distinct fragmented peptides (p < 0,05)
V1	8615601	**cyclase [Vitis pseudoreticulata]**	532	ProteinPilot	14	EFESDYAGFTEDGAR
						EVILVESLK
						KEFESDYAGFTEDGAR
						LDDVPAGMYNVHCLHLR
						LPGAEGAPIR
						SEAYPSAYGSGSCNVELIPVKR
						WLVENTDIK
						EFESDYAGFTEDGAR
						GPALLVDAPR
						LPGAEGAPIR
V2	49388156	**putative chlorophyll a/b-binding protein type III precursor [Oryza sativa Japonica Group]**	270	ProteinPilot	10.67	FQDWANPGSMGK
						QGADRPLWFASK
						QSLTYLDGSLPGDYGFDPLGLSDPEGTGGFIEPR
						QYFLGLEK
						WLAYGEVINGR
						RFQDWANPGSMGK
						LKEVKNGR
						QGADRPLWFASK
						QYFLGLEK
						RFQDWANPGSMGK
						WLAYGEVINGR
V3	225446775	**oxygen-evolving enhancer protein 2, chloroplastic [Vitis vinifera]**	449	ProteinPilot	2.13	SITDYGSPEEFLSK
						TNTDFLPYNGEGFK
						EFPGQVLR
V4	73647738	**ascorbate peroxidase [Vitis pseudoreticulata]**		ProteinPilot	9.32	ALLSDPAFRPLVEK
						EDKPEPPPEGR
						NCAPIMLR
						SYPTVSEEYKK
						TGGPFGTMK
						EDKPEPPPEGR
						NCAPIMLR
V5	349048	**ribulose-1,5-bisphosphate carboxylase/oxygenase large subunit, partial (chloroplast) [Pogostemon cablin]**		MASCOT	121.49	TFKGPPHGIQVER
V6	225460496	**PREDICTED: ATP synthase delta chain, chloroplastic [Vitis vinifera]**		MASCOT	154.41	LESEHLAQIAK
						TAIDPSLVAGFTIR
						EIAKEFELVYNR
V7	225461287	**PREDICTED: cytochrome b6-f complex iron-sulfur subunit, chloroplastic isoform 1 [Vitis vinifera]**		MASCOT	234.97	GDPTYLVVENDK
						DALGNDVIADEWLK
						FICPCHGSQYNNQGR
V8	22797822	**ATP synthase epsilon subunit [Vitis vinifera]**		MASCOT	258.01	TRVEAINVTS
						QIIEANLALR
						IGNNEITVLVNDAEK
						LNDQWLTMALMGGFAR
V9	359475330	**PREDICTED: glycine-rich RNA-binding protein GRP1A-like [Vitis vinifera]**		MASCOT	313.75	DRGYGDGGSR
						NITVNEAQSR
						AFSQFGEILESK
						GGGGGYGGGGGGYGGGSR
						GFGFVTFSSEQSMR
						CFVGGLAWATDDQSLER
V10	225468761	**oxygen-evolving enhancer protein 1, chloroplastic [Vitis vinifera]**	610	MASCOT	984.17	VPFLFTIK
						RLTYDEIQSK
						FGGEFLVPSYR
						FCLEPTSFTVK
						KFCLEPTSFTVK
						DGIDYAAVTVQLPGGER
						GTGTANQCPTIDGGVDSFAFK
						FEEKDGIDYAAVTVQLPGGER
						SKPETGEVIGVFESIQPSDTDLGAK
V11	225459768	**plastocyanin, chloroplastic isoform 1 [Vitis vinifera]**	331	MASCOT	453.18	GTYSFYCSPHQGAGMVGK
						ISMSEEDLLNAPGEVYSVTLTEK
						NNAGFPHNVVFDEDEVPSGVDVSK
V12	30687535	**Quinone reductase family protein [Arabidopsis thaliana]**	393	MASCOT	62.96	AFLDATGGLWR
V13	225456238	**PREDICTED: glutamine synthetase cytosolic isozyme 1[Vitis vinifera]**		MASCOT	183.79	VIVEYIWVGGSGMDLR
						GNNILVMCDTYTPAGEPIPTNKR
V14	359473178	**Quinone oxidoreductase-like protein At1g23740, chloroplastic-like [Vitis vinifera]**	612	MASCOT	460.41	VKPVVDPK
						LNPYLESGK
						KLNPYLESGK
						VVAAALNPVDAK
						AWVYGDYGGVDVLK
						QFGSFAEYTAVEEK
						EGGSVVALTGAVTPPGFR
						ELKEGDEVYGDINEK
						ATDSPLPTVPGYDVAGVVVK
V15	225432496	**PREDICTED: glutamine synthetase leaf isozyme, chloroplastic [Vitis vinifera]**		MASCOT	349.06	DISDAHYK
						AAEIFGNKK
						EHISAYGEGNER
						TISKPVEHPSELPK
						HKEHISAYGEGNER
						HETANINTFSWGVANR
						GGNNILVICDSYTPAGEPIPTNKR
S1	4838443	**symbiosis regulated acidic polypeptide SRAP32-3 [Pisolithus tinctorius]**		Protein Pilot	4	DKLEAKLDKAAGDYIDGVDI
						TDVANSLEFASR
S2	160897637	**hypothetical protein Daci_2194 [Delftia acidovorans SPH-1]**		MASCOT	59.58	ERAQSAAAIER
S3	71659717	**hypothetical protein [Trypanosoma cruzi strain CL Brener]**		MASCOT	58.93	KDIAEEVLER
S4	20162432	**AF493154_1 32 kDa-cell wall symbiosis regulated acidic polypeptide [Pisolithus microcarpus]**		MASCOT	80.35	NDPLYSEAEK
S5	71659717	**hypothetical protein [Trypanosoma cruzi strain CL Brener]**		MASCOT	57	KDIAEEVLER
S6	20162434	**32 kDa-cell wall symbiosis regulated acidic polypeptide precursor [Pisolithus microcarpus]**	358	MASCOT	210.62	ATGDDYAR
						NSLEFAAR
						FQLAVCSEK
						AADKATGDDYAR
S7	390601324	**cysteine peroxiredoxin [Punctularia strigosozonata HHB-11173SS5]**	391	MASCOT	334.84	NFDEVLR
						TVFVIDPK
						LTISYPASTGR
						VVDSLQLGDKYR
						LGSIAPDFEAETTAGPIK
						ISTLYDMLDEQDATNR
S8	225461209	**PREDICTED: flavoprotein wrbA isoform 1 [Vitis vinifera]**		MASCOT	383.52	GAASVEGVEAK
						KGAASVEGVEAK
						AFLDATGGLWR
						GGSPYGAGTFAGDGSR
						VKGGSPYGAGTFAGDGSR
						VYIVYYSMYGHVEK
S9	20097	**jgi|Pisti1|20097|gm1.2716_g**		MASCOT	54.44	NPDIQAPR
S10	218533914	**serine proteinase inhibitor [Clitocybe nebularis]**	50.1	MASCOT	119.09	AQEWVIR
						YRELQDAYTIVK
S11	20097	**jgi|Pisti1|20097|gm1.2716_g**		MASCOT	302.72	VFAVMEGR
						LDEPGEIGWIAPTDGSSQIR
						RLDEPGEIGWIAPTDGSSQIR
						EIPTAPPGQYRPEELYNLAFPLE
S12	218533914	**serine proteinase inhibitor [Clitocybe nebularis]**	50.1	MASCOT	89.1	AQEWVIR
						ELQDAYTIVK
						YRELQDAYTIVK
S13	20097	**jgi|Pisti1|20097|gm1.2716_g**		MASCOT	343.22	LDEPGEIGWIAPTDGSSQIR
						RLDEPGEIGWIAPTDGSSQIR
						EIPTAPPGQYRPEELYNLAFPLE
S14	33323059	**major latex protein [Ficus pumila var. awkeotsang]**	187	MASCOT	485.65	GIDEHITKA
						LREDVPAPDK
						EKVEYDDANR
						SPPEKYYNIFK
						SATLIGVDGDIMQEYK
						GQAYHVPNAAPDHIQGVDVHEGDWETHGSVK
S15	3164115	**major latex-like protein [Rubus idaeus]**		MASCOT	68.2	EKVELDDVNK
S16	Q9S1X8	**Na(+)/H(+) antiporter NhaA 1/4[Streptomyces coelicolor strain ATCC BAA-471/A3(2)/M145]**		MASCOT	483.67	NDAYVIAK
						EEREEER
						GVGWVAPSPENK
						VGECTYVISAR
						SVTEPPTFNMEK
						KSVTEPPTFNMEK
						GVGWVAPSPENKEER
S17	375333787	**lectin 2 [Agrocybe aegerita]**	565	MASCOT	647.19	FLGEATGDGR
						FVVDLTGDGR
						DFAYSAGGWR
						DGFSIQPFVAIK
						ADIVGFGDGGVLVSK
						SVIDNFTYSAGGWR
						FVLNNFGVQQGWQVNK
						NTGGGNFSPASLALNDFGYNAGGWR
S18	392590852	**phosphoglycerate mutase-like protein [Coniophora puteana]**	540	MASCOT	431.54	VYASPEFK
						DIGGIGNLPGR
						TAQPFFGAIR
						LPPTLIEQAR
						GPAPEDRDFLR
						ADIPLTEFFYR
						SVYLSPSSPSYITNMK
S19	160184939	**Serine protease inhibitor [Lentinula edodes (Shiitake mushroom)]**	58.9	MASCOT	106.97	WCIQYTER
						VGDCTYVISAR
S20	1001331	**jgi|Pisti1|1001331|fgenesh1_kg.33_#_73_#_Locus10529v3rpkm0.40_PRE**		MASCOT	205.69	YYINYLIER
						WIITFVPQPGR
						NNLLYEQVTAPQK
P1	332591479	**phosphoglycerate kinase 1 [Pinus pinaster]**		MASCOT	260.9	AHASTEGVTK
						LTELLGVNVVK
						ELDYLVGAVSNPK
						ADLNVPLDENQNITDDTR
P2	396547	**glutamate-ammonia ligase [Pinus sylvestris]**		MASCOT	134.75	SLSGPVSSVK
						VIAEYIWIGGSGMDMR
P3	218155	**chloroplastic aldolase [Oryza sativa Japonica Group]**		MASCOT	129.98	EAAWGLAR
						AKANSLAQLGK
						LASIGLENTEANR
P4	3415126	**phenylcoumaran benzylic ether reductase [Pinus taeda]**		MASCOT	497.86	VVILGDGNAR
						SLAQAGLTAPPR
						ILLIGATGYIGR
						DKVVILGDGNAR
						ASLDLGHPTFLLVR
						FFPSEFGNDVDNVHAVEPAK
						GDQTNFEIGPAGVEASQLYPDVK
						AIEAEGIPYTYVSSNCFAGYFLR
P5	413951269	**ferredoxin-NADP reductase, leaf isozyme [Zea mays]**	768	MASCOT	388.09	KDNTYVYMCGLK
						RLVYTNDQGEIVK
						LYSIASSALGDFGDSK
						ITGDDAPGETWHMVFSTEGEIPYR
P6	359473184	**carbonic anhydrase, chloroplastic-like isoform 2 [Vitis vinifera]**	299	MASCOT	109.34	FMVVACADSR
						QTAFIEDWIK
P7	359473184	**carbonic anhydrase, chloroplastic-like isoform 2 [Vitis vinifera]**	299	MASCOT	107.77	FMVVACADSR
						QTAFIEDWIK
P8	14719331	**putative 3-beta hydroxysteroid dehydrogenase/isomerase protein [Oryza sativa]**	496	MASCOT	245.13	MKPGFDPSK
						IGGGDDVFVGDIR
						AEQYLADSGLPYTIIR
						KAEQYLADSGLPYTIIR
P9	116790330	**unknown [Picea sitchensis]**		MASCOT	104.32	TTFLSDSEVK
						TTFLSDSEVKR
P10	116782111	**unknown [Picea sitchensis]**		MASCOT	220.45	EYYNISVLTR
						YEDNGDTVSNVSVMVIPTDKK
P11	16798638	**AF434186_1 Cu-Zn-superoxide dismutase precursor [Pinus pinaster]**		MASCOT	234.71	LTHGAPEDDVR
						KLTHGAPEDDVR
						GGHELSLTTGNAGGR
						GNSQVEGVVNLSQEDNGPTTVK
P12	2911276	**LMW heat shock protein [Fragaria x ananassa]**	103	MASCOT	105.95	QPEPQPPQPK
						ASMEDGVLTVTVPK
P13	413946843	**Putative peptidyl-prolyl cis-trans isomerase family protein [Zea mays]**	307	MASCOT	138.86	TFEDENFK
						KLESEETNR
						IVLGLFGEDVPK
P14	20794	**Type III chlorophyll a/b-binding protein [Pinus sylvestris]**	259	MASCOT	268.1	LQDYRNPGSMGK
						YLGGSGNPAYPGGPLFNPLGFGK
						YLGGSGNPAYPGGPLFNPLGFGKDEK

(Protein annotations retrieved from NCBI protein database restricted to **Viridiplantae**, to ***Vitis*, to Agaricomycotina**, **JGI*Pisolithus tinctorius*manual** and NCBI **Blastp**).

## Discussion

Woody plant tissues contain significant amounts of secondary metabolites with different roles ranging from structural functions to defence against pathogens (Rhodes 1994). Most plant secondary metabolites belong to the class of phenolics including phenols, flavonoids, stilbenes, terpenes, tannins and lignins (Rhodes 1994) and can negatively interfere with protein extraction and 2-DE protein separation. For example, phenolics can build irreversible complexes with proteins, and the oxidation of phenolics by phenoloxidases and peroxidases can cause streaking and generate artifactual spots on gels (Vâlcu and Schlink 2006). Carbohydrates can block gel pores causing precipitation and extended focusing times, resulting in streaking and resolution loss (Carpentier et al. 2005). Also terpenoids, pigments, lipids and waxes produce streaking and charge heterogeneity (Carpentier et al. 2005). Secondary metabolites accumulate as soluble forms in the vacuoles and are more abundant in adult mature tissues than in young etiolated tissues (Granier 1988). Thus, sample preparation becomes a critical step for a proteomic approach focused on mature woody plants tissues. In the context of proteomic studies, comparison of 2-DE gels requires well-resolved proteomes. For total proteome extraction, an ideal protocol should reproducibly capture all the protein species composing the proteome with low contamination from other molecules. In the present study, the protocols based on ethanol-acetone (Ferreira et al. 2006), TCA-acetone (Damerval et al. 1986), and phenol (Hurkman and Tanaka 1986) were evaluated for proteome isolation, on three different woody recalcitrant plant tissues: grapevine leaves, pine needles and ECM oak roots. To compare the effects of ethanol, phenol and TCA protein extraction methods on the 2-DE maps, equal amounts of protein extracted from the different plant materials, were separated by 2-DE under identical conditions. Comparison of the extraction methods was done based on protein yield, spot focusing and resolution. Additionally, several 2-DE protein spots from each of the species/tissues analyzed were selected from gels of the best performing method, phenol extraction, to evaluate its compatibility and quality for protein identification by MS-based techniques.

Considering protein yield, TCA-acetone and ethanol precipitation methods produced higher yields than the phenol method for all the species/tissues analyzed. Studies comparing the performance of TCA and phenol protocols have been conducted earlier by Saravanan and Rose (2004) and Carpentier et al. (2005), that reported the same protein yield by the two methods in several recalcitrant fruit tissues (tomato, orange, banana and avocado), leaves and roots. However, the tissues analyzed in our study are much more lignified than the ones used by these authors and this could have contributed to the observed difference in protein yield between the two extraction protocols. Leaves and roots of woody plants are very rich in lignin, an aromatic polymer that results from the oxidative combinatorial coupling of 4-hydroxyphenylpropanoids which accumulates in the walls of secondary thickened cells, causing rigidness (Vanholme et al. 2010). We hypothesize that these compounds, present in our samples, could have co-precipitate with proteins in the TCA and ethanol protocols leading to an overestimation of protein yield using the Bradford assay. The Coomassie blue dye in this assay binds primarily to aromatic amino acid residues (Bio-Rad Protein Assay Manual), possibly also binding to the aromatic compounds of lignin leading to false positive results in woody plant tissues. This is corroborated by the observation in our samples of a lower spot number in 2-DE gels from the TCA and ethanol protocols, when compared with the phenol protocol (Figure 1). A similar result was also reported in a study comparing TCA and phenol protein extraction of Douglas fir needles, a woody plant tissue like the ones hereby analysed, with TCA showing lower intensity spots when compared to gels from a phenol protocol (Dziedzic and McDonald 2012). TCA has been reported as a suitable extraction method for soft/young plant tissues but it was found unsuitable for more complex plant tissues due to the co-extraction of polymeric contaminants (Saravanan and Rose 2004;Carpentier et al. 2005). Using the phenol protocol, similar protein yields were obtained to the ones reported for other woody plant tissues (Wang et al. 2003,2006;Dziedzic and McDonald 2012) extracted with a phenol based protocol, corroborating our results. As expected, protein recovery from roots was substantially lower than from leaves/needles, for the three protocols used, highlighting the cellular structural differences between the two tissues. Roots are highly vacuolated tissues containing lower protein amounts when compared to aerial parts, which makes them one of the most recalcitrant plant tissues for protein purification.

For the three species/tissues analyzed, the phenol extraction protocol produced the best quality gels despite presenting the lowest protein yields. The phenol 2-DE gels showed higher number of spots, increased resolution and spot focusing, increased number of high molecular weight spots, and lower background when compared with TCA-acetone and ethanol-acetone methods. Using the phenol extraction, up to 904, 805 and 532 spots were resolved from ECM oak roots, pine needles and grapevine leaves, respectively. These values are in agreement with the number of spots obtained in the same species/tissues previously reported (Burgess et al. 1995;Jellouli et al. 2010;Liu et al. 2012).

Phenol has been reported as the most suitable protein extraction protocol for tissues containing low concentrations of protein and high content of interfering compounds that inhibit electrophoresis (Saravanan and Rose 2004;Wang et al. 2008). It has been widely used to extract proteins from difficult plants like olive and cotton (Wang et al. 2003;Yao et al. 2006), or fruits including banana, strawberry, apple or grape (Saravanan and Rose 2004;Vincent et al. 2006;Wang et al. 2008). Its superior performance has been attributed to a higher capacity to physically separate proteins from contaminating substances like nucleic acids, carbohydrates and cellular debris. Therefore, a great amount of the 2-DE interfering substances are immediately eliminated in the aqueous phase through phase separation, which is increased by the presence of added sucrose. Proteins, which remain solubilized and mostly purified in the phenolic phase, can then be precipitated with methanol and ammonium acetate (Faurobert et al. 2007). In addition to its selectivity as a solvent, phenol is one of the strongest dissociating agents known to decrease molecular interactions between proteins and other materials (Carpentier et al. 2005).

In order to determine the compatibility of the phenol isolated proteome from the species/tissues analysed with protein identification methods, several protein spots were excised from 2-DE gels and subjected to MS analysis. Identification of all the excised spots confirmed the compatibility of the phenol extraction protocol with MS protein identification. This is in agreement with previous studies on protein extraction from recalcitrant fruit tissues (Carpentier et al. 2005;Zheng et al. 2007) and woody plant tissues (Wang et al. 2003,2006;Dziedzic and McDonald 2012). Some of the proteins identified, such as SRAP32 from *P. tinctorius* identified in oak ECM roots, were previously described (Burgess et al. 1995;Laurent et al. 1999) in the symbiotic roots of other forest tree species. These acidic cell wall symbiosis regulated proteins (SRAPS) are induced by ECM development and are thought to be involved in the attachment of fungal hyphae to the root surface during symbiosis formation. In our 2-DE gels, SRAP32 molecular mass and isoelectric point is in accordance to those reported earlier (Burgess et al. 1995;Laurent et al. 1999). Also, for ECM cork oak roots only 3 out of the 20 protein spots analysed match plant proteins, which is in accordance to Burgess et al. (1994) and Zeppa et al. (2005), which report a marked inhibition of the plant polypeptide synthesis and an enhanced accumulation of fungal peptides during ECM development. For grapevine leaves and pine needles, several photosynthesis/energy related proteins, such as ribulose-1,5-bisphosphate carboxylase/oxygenase large subunit, chloroplastic aldolase or ATP synthase delta chain chloroplastic, among others were identified, which is in agreement with the photosynthetic and carbon fixation primary function of foliar tissues. Photosynthesis and energy related proteins were also the major group of proteins identified by ESI-MS/MS in Douglas-fir needles (Dziedzic and McDonald 2012).

## Conclusions

The phenol extraction protocol allowed an efficient proteome isolation and 2-DE separation of the woody recalcitrant plants used in this study. Also, the resulting protein spots were found to be compatible with identification by MALDI-TOF/TOF. This study illustrates the need to establish a proper protein extraction method when preparing plant tissues for proteomic analysis, particularly when working with woody recalcitrant plant tissues containing high levels of interfering compounds.

## Methods

### Plant material

#### Grapevine

*V. vinifera* ‘Regent’ grapevine wood cuttings were harvested at Quinta da Plansel (Montemor, Portugal) and grown in 12 cm ø pots under greenhouse conditions (natural day/night rhythm and a temperature range between 5 and 28°C) for ten weeks. Leaves were harvested, frozen and grounded in liquid nitrogen using a mortar and pestle and stored at −80°C until protein extraction.

### Pine

*Pinus pinaster* trees with breast height diameter (BHD) classes > 20 cm were selected in mid-end June (Comporta, Portugal). Samples were collected from one branch of the lower canopy at a height of at least 8 m. Needles were harvested, frozen in liquid nitrogen and stored at −80°C.

### Cork oak

The *Pisolithus tinctorius* (Pers.) Couker & Couch isolate Pt23 from the collection of the Center of Biodiversity, Functional & Integrative Genomics (BioFIG), Sciences Faculty of Lisbon University, was grown on a peat/vermiculite (v/v) mixture moistened with liquid BAF medium (Moser 1960), for two months in the dark at 25°C, and then used as ECM inoculum. *Quercus suber* L. seeds were surface disinfected by shaking in 30% commercial bleach for 30 min and washing in four changes of distilled water. Seeds were sown on soil in plastic trays, and seedlings were grown in a greenhouse under natural light and temperature and watered as needed. Four months old seedlings were transferred from the sowing beds to 1,5 L pots containing soil, and inoculated with the fungal inoculum by depositing 350 mL of peat-vermiculite grown mycelium (previously rinsed with water to remove excess nutrients) in the plantation hole, in direct contact with the roots. Four months after inoculation, ten cork oak ectomycorrhizal seedlings were sampled. Roots were rinsed to eliminate soil particles, first with tap water and after with deionized water. Excess water was removed with filter paper. Secondary roots presenting ECM root tips were sampled and immediately frozen in liquid nitrogen, grounded and stored at −80°C.

### Proteome extraction

#### Ethanol-acetone method

Plant tissue (1 g) was dispersed in 4 vol of ethanol (Merck). After 1 h at −20°C, the same volume of cold acetone (Merck) was added and proteins were allowed to precipitate overnight, at −20°C. Proteins were collected through centrifugation at 26000 g (−10°C, 15 min), followed by a washing step with ethanol:acetone:triple distilled water 4:4:1 (v/v/v) with 9 sample volume for 6 h at −20°C. Proteins were recovered by centrifugation at 26000g (−10°C, 40 min), followed by two additional washing steps. The final pellet was dried overnight at room temperature and solubilized in lysis buffer [7 M urea, 2 M thiourea, 0.25% (v/v) of Pharmalyte 3–10 and 0.5% (v/v) of Pharmalyte 4–7 (Amersham Pharmacia Biotech, Uppsala, Sweden), 2% (w/v) 3-[(3-Cholamidopropyl) dimethylammonio]-1-propanesulfonate (CHAPS) and 25 mM dithiothreitol (DTT)] for 24 h at room temperature. Protein quantification was performed with Bradford reagent (Bradford 1976) using Bovine Serum Albumin (BSA) as standard (Bio-Rad protein assay, BioRAD, USA). Solubilized proteomes were kept at −20°C until further use. Three technical replicates of each extraction were performed for each species.

### TCA-acetone method

Plant tissue (1 g) was suspended in 10% TCA (w/v) (Sigma) in acetone (Merck) at −20°C, with 0.1% (w/v) of DTT (Sigma). Proteins were precipitated overnight at −20°C and recovered through centrifugation at 26000 g for 1 h at −10°C. Pellet was resuspended in 90% (v/v) acetone at −20°C with 0.1% (w/v) DTT and precipitated for 2 h at −20°C, followed by centrifugation at 26000 g for 45 min at −10°C. This washing procedure was repeated twice. Final protein solubilisation and quantification procedures were done as described above. Three technical replicates of each extraction were performed.

### Phenol extraction method

Plant tissue (1 g) was suspended in 10 mL of extraction buffer [5 mL of Tris pH 8.8 buffered phenol and 5 mL of extraction media (0.1 M Tris–HCl pH 8.8, 10 mM EDTA, 0.4% (w/v) 2-mercaptoethanol and 0.9 M sucrose)]. Samples were homogenized and incubated for 30 min at 4°C with agitation and then centrifuged 10 min at 5000 g, 4°C. The phenol phase was recovered and proteins were precipitated by addition of 5 vol of 0.1 M ammonium acetate in 100% methanol (pre-chilled to −20°C) and incubated overnight at −20°C. The precipitate was collected by centrifugation (30 min, 4000 g, -10°C) washed twice with the ammonium acetate solution in methanol, twice with ice-cold 80% (v/v) acetone and one time with cold 70% (v/v) ethanol. Between each washing step, the resuspended sample was kept at −20°C for 20 min. Final protein solubilization and quantification procedures were done as described above. Three technical replicates of each extraction were performed for each species.

### Two-dimensional electrophoresis

Analytical gels were performed using 18 cm IPG strips of linear 4–7 pH gradient (GE Healthcare). Prior proteins isoelectric focusing (IEF), strips were passively rehydrated overnight with lysis buffer containing 300 μg of protein per sample in an IEF Rehydration Tray (GE Healthcare). IEF was performed using an IPGphor™ Isoelectric Focusing System (Amersham-Pharmacia Biotech Pharmacia Biotech) with the IPGPhor Manifold. IEF was performed for 26 h at 20°C to a total of 86000 Vh. Subsequently, focused IPG strips were immediately equilibrated for 15 min in equilibration buffer [2% (w/v) sodium dodecyl sulfate (SDS), 10% (v/v) glycerol, 50mM Tris–HCl pH 6.8 and 1% (v/v) DTT], followed by immediate storage at −80°C until use, as previously described (Ferreira et al. 2006). IPG strips were thawed and reequilibrated for 15 min using fresh equilibration buffer (Ferreira et al. 2006), and immediately loaded onto 26 × 20 × 0.1 cm^3^ 15% polyacrylamide gels (acrylamide:bisacrylamide at 200:1). The top of the gel was sealed using agarose sealing solution (0.5% (w/v) agarose in running buffer with bromophenol blue). Electrophoresis was performed in recirculating running buffer for 16 h at 10°C, under constant power settings (80 mA). The three replicates prepared per extraction protocol were resolved on two-dimensional polyacrylamide gel electrophoresis (2D-PAGE). 2D-PAGE was allowed to run until the dye front reached the lower end of the gels. Protein isoelectric points were determined by the use of Isoelectric Focusing Calibration kit Broad p*I* (pH 4–7), while their molecular masses were determined using PageRuler™ unstained protein ladder (Thermo Fisher Scientific). Gels were stained with Oriole™ fluorescence gel stain (Bio-Rad), following manufacturer’s instructions. Given the broad UV excitation of Oriole™, image acquisition was done on the UV-based image equipment ChemiDoc™ X^RS+^ (BioRad) using the software Image Lab™ 2.0. Gels exposure times to UV excitation were always set below the limit of spot saturation.

### Image analysis

The 2-DE gel images were analyzed using REDFIN software v. 3.3 (http://www.ludesi.com). Each protein extraction method (TCA-acetone, phenol and ethanol-acetone) was represented by three 2-DE gels images matching three technical replicates. For each protocol, gel images were warped after setting vector points to construct a composite image (i.e. raw master gel). This fusion gel image, i.e. normalized image, was created to eliminate noise and minor discrepancies between gels. The spots were detected and quantified as the cumulative intensity of optical density of each spot, proportional to spot volume. Normalization of spot volumes was automatically done by REDFIN 3 software (Ludesi, Lund, Sweden, http://www.ludesi.com) using the total spot volume methods, by removing technical differences in staining, scanning and sample volume. Spot-by-spot visual validation of automated analysis was done thereafter to increase the reliability of the matching (Chich et al. 2007). Experimental p*I* was determined using a 4–7 linear scale over the total length of the IPG strip (18 cm). M_*r*_ values were calculated by mobility comparisons with the PageRuler™ protein ladder (Thermo Fisher Scientific). Total number of spots was calculated as spots present in three technical replicate gels.

### MS analysis and protein identification

Preparative 2-DE gels loaded with 600 μg of protein extracted with the phenol-based method, for each plant were used for spot picking. After 2-DE, the gel was colloidally CBB-stained (Neuhoff et al. 1988) and around 2% (52 spots) of total spots present per plant material (15 spots on grapevive leaves, 15 spots for pine needles and 22 for oak ECM roots) were randomly excised and trypsin-digested as described by da Costa et al. (da Costa et al. 2008). Sample peptides were acidified with formic acid, desalted, and concentrated with POROS R2 microcolumns (Applied Biosystems, Foster City, CA) and co-crystallised in MALDI-TOF/TOF sample plates according to da Costa et al. (da Costa et al. 2008) using the matrix α-cyano-4-hydroxycinnamic acid (CHCA). Tandem MS/MS was performed using a MALDI-TOF/TOF 4800 plus MS/MS (Applied Biosystems, Foster City, CA, USA). The MS/MS was externally calibrated using des-Arg-Bradykinin (904.468 Da), angiotensin 1 (1296.685 Da), Glu-Fibrinopeptide B (1570.677 Da), ACTH (1–17) (2093.087 Da), and ACTH (18–39) (2465.199 Da) (4700 Calibration Mix, Applied Biosystems, Foster City, CA, USA). Each reflectron MS spectrum was collected in a result-independent acquisition mode, typically using 1000 laser shots per spectra and a fixed laser intensity of 3500V. The fifteen strongest precursors were selected for MS/MS, the weakest precursors being fragmented first. MS/MS analyses were performed using CID (Collision Induced Dissociation) assisted with air, with a collision energy of 1 kV and a gas pressure of 1 × 10^-6^ torr and the PRIDE Team for all the support during data submission to the public data repository PRoteomics IDEntifications database PRIDE. Two thousand laser shots were collected for each MS/MS spectrum using a fixed laser intensity of 4500V.

Protein identification was performed by homology search on different protein databases using the Mascot and Protein Pilot (Applied Biosystems, Foster City, CA, USA) search engines. Searches in MASCOT (v. 2.2; Matrix Science, Boston, MA, USA) were performed without taxonomical restrictions, a minimum mass accuracy of 30 ppm for the parent ions, an error of 0.3 Da for the fragments, trypsin as digesting enzyme with one missed cleavage allowed, and carbamidomethylation of Cys and oxidation of Met as fixed and variable amino acid modifications, respectively. ProteinPilot (Protein Pilot software v. 3.0, rev. 114732; Applied Biosystems, Foster City, CA, USA) searches were performed without taxonomic restrictions and search parameters set as follows: enzyme, trypsin; Cys alkylation, iodoacetamide; special factor, gel-based ID; and ID focus, biological modification and amino acid substitution. Peptide sequences belonging to the different plant species, i.e. grapevine and pine leaves, and ECM oak roots, were queried against NCBI’s Viridiplantae protein database available on both in-house Mascot and ProteinPilot servers. The NCBI proteins from *Vitis* (102484 entries, July 2012) and Agaricomycotina (334526 entries, July 2012), and the proteins from *P. tinctorius* Marx 270 v1.0 at the JGI portal (BestModels v1.0, release date April 10, 2012; http://genome.jgi-psf.org/Pisti1/Pisti1.home.html) were also queried for annotation. Protein sequences that were identified as “unknown” or as “hypothetical protein”, were further annotated by using the protein homologs sequences for an additional query using BLASTP algorithm (http://blast.ncbi.nlm.nih.gov/Blast.cgi), searching first the UniProtKB/Swiss-Prot database, and then the NCBI non redundant database. The mass spectrometry proteomics data have been deposited to the ProteomeXchange Consortium (http://proteomecentral.proteomexchange.org) via the PRIDE partner repository (Vizcaíno et al., 2013) with the dataset identifier PXD000224.

## Electronic supplementary material

Additional file 1: Table S1: Complete information of the identified peptides per protein from cork oak ectomycorrhizal roots spots. (XLSX 67 KB)

Additional file 2: Table 2: Complete information of the identified peptides per protein from pine needles spots. (XLSX 49 KB)

Additional file 3: Table 3: Complete information of the identified peptides per protein from grapevine mature leaves spots. (XLSX 58 KB)
